# Anxiety in Relatives of Patients Admitted to Cardiac Care Units and its Relationship with Spiritual Health and Religious Coping

**DOI:** 10.17533/udea.iee.v38n3e10

**Published:** 2020-11-11

**Authors:** Fereshteh Dehghanrad, Marjan Mosallanejad, Marzieh Momennasab

**Affiliations:** 1 M.Sc. Instructor. Email: dehghanrad@sums.ac.ir dehghanrad@sums.ac.ir; 2 Master student of critical care. Email: marjan.6666@yahoo.com marjan.6666@yahoo.com; 3 Ph.D. Of Nursing, Associate Professor. Email: Momennasab@sums.ac.ir. Corresponding author Momennasab@sums.ac.ir; 4 Department of Nursing, School of Nursing and Midwifery, Shiraz University of Medical Sciences, Shiraz, Iran. Shiraz University of Medical Sciences Shiraz Iran

**Keywords:** family, anxiety, spirituality, adaptation, psychological, coronary care units, cross-sectional studies, familia, ansiedad, espiritualidad, adaptación psicológica, unidades de cuidados coronarios, estudios transversales, família, ansiedade, espiritualidade, adaptação psicológica, unidades de cuidados coronarianos, estudos transversais

## Abstract

**Objective.:**

This work sought to determine the level of anxiety in relatives of patients admitted to CCUs and its relationship with spiritual health and religious coping.

**Methods.:**

This cross-sectional study was conducted on 300 relatives of Cardiac Care Units patients in Jahrom, Iran. Required data was collected using the Spielberger State-Trait Anxiety Inventory (STAI), the Paloutzian-Ellison Spiritual Well Being Scale (SWBS), and the Pargament Brief RCOPE questionnaire.

**Results.:**

The results showed that both levels of state and trait anxiety were moderate and the level of total spiritual health was high. Anxiety score had an inverse relationship with spiritual health (r=-0.52) and a direct relationship with negative religious coping score (r=0.25). However, no significant relationship was found between total anxiety score and positive religious coping (*p*>0.05). There was a direct relationship between spiritual health and positive religious coping (r=0.19), and an inverse relationship between spiritual health and negative religious coping (r=-0.36).

**Conclusion.:**

According to the findings of the study, it is suggested to paying attention to the reinforcement of spiritual attitudes, beliefs, and religious coping strategies to reduce their anxiety in CCU patients.

## Resumen

**Objetivo.:**

Este trabajo buscó determinar el nivel de ansiedad en familiares de pacientes ingresados ​​en Unidades de Cuidados Cardíacos -UCC- y su relación con la salud espiritual y el afrontamiento religioso.

**Métodos.:**

Este estudio transversal se llevó a cabo en 300 familiares de pacientes hospitalizados en UCC en Jahrom, Irán. Los datos requeridos se recopilaron utilizando el Inventario de Ansiedad - Estado - Rasgos (STAI) de Spielberger, la Escala de Bienestar Espiritual de Paloutzian-Ellison (SWBS) y el cuestionario RCOPE en versión breve de Pargament.

**Resultados.:**

Los hallazgos mostraron que tanto los niveles de ansiedad del estado como de los rasgos eran moderados y el nivel de salud espiritual total era alto. La puntuación de ansiedad tuvo una relación inversa con la salud espiritual (r=-0.52) y una relación directa con la puntuación de afrontamiento religioso negativo (r=0.25). Sin embargo, no se encontró una relación significativa entre la puntuación total de ansiedad y el afrontamiento religioso positivo (*p*>0.05). Hubo una relación directa entre la salud espiritual y el afrontamiento religioso positivo (r=0.19), y una relación inversa entre la salud espiritual y el afrontamiento religioso negativo (r=-0.36).

**Conclusión.:**

De acuerdo con los hallazgos del estudio, se sugiere prestar atención al refuerzo de las actitudes espirituales, creencias y estrategias de afrontamiento religiosas para reducir su ansiedad en los pacientes con CCU.

## Resumo

**Objetivo.:**

Este trabalho buscou determinar o nível de ansiedade em familiares de pacientes internados em Unidades de Cuidados Cardíaco - UCC- e sua relação com a saúde espiritual e o enfrentamento religioso.

**Métodos.:**

Este estudo transversal foi realizado em 300 familiares de pacientes internados na UCC em Jahrom, no Irão. Os dados necessários foram coletados usando o Spielberger Trait Anxiety Inventory (STAI), a Escala Paloutzian-Ellison de Bem-estar Espiritual (SWBS) e o questionário RCOPE de Pargament.

**Resultados.:**

Os resultados mostraram que tanto os níveis de ansiedade do estado como os traços eram moderados e o nível geral de saúde espiritual era alto. A pontuação de ansiedade teve uma relação inversa com a saúde espiritual (r=-0.52) e uma relação direta com a pontuação de afrontamento negativo religioso (r=0.25). No entanto, não foi encontrada relação significativa entre a pontuação total de ansiedade e o afrontamento religioso positivo (*p*>0.05). Houve relação direta entre saúde espiritual e enfrentamento religioso positivo (r=0.19), e relação inversa entre saúde espiritual e enfrentamento religioso negativo (r=-0.36)

**Conclusão.:**

De acordo com as descobertas do estudo, sugere-se colocar atenção para o reforço de atitudes espirituais, crenças e estratégias religiosas de enfrentamento para reduzir sua ansiedade em pacientes com CCU.

## Introduction

Cardiac disease is the leading global leading cause of death and disability.(1) Coronary artery disease (CAD) is the most common type of heart disease and is more common in older patients.(2) It is expected that disability adjusted life year lost due to cardiovascular-related premature death increase twofold by 2025.(3) Admission to Cardiac Care Units **(**CCU) is viewed as a crisis for both the patients and their family members since they are suddenly pushed into an unknown situation of exposure with a critical disease and its stressful outcomes.(4) Family members of patients often suffering from physical and mental health problems.(5). The stressful critical care unit environment, critically ill patients who need immediate specialized monitoring, high mortality risk, complex technical and medical equipment, permanent monitoring of patients, alarm sounds, and restricted visiting hours are the factors affecting anxiety in relatives of critical care patients.(6) Some studies have shown that anxiety level in family members and first-degree relatives of CCU patients is equal to the anxiety level of the patients themselves.(7)

The prevalence of anxiety varied from 15% to 24% in caregivers after discharge of their patients from the intensive care unit.(8) A significant number of family members of ICU patients experience moderate to severe anxiety.(9) Experts believe that paying attention to spirituality is among the effective factors in reducing anxiety.(10) Spirituality constitutes a dimension of health alongside physical, mental, and social dimensions.(11) Spirituality is characterized by stability in life, peace, and feeling close relationship with himself, God, society, and the environment.(12)

People with higher level of spirituality are more resistant to illness and resilient to stress.(13) Spirituality is an efficient coping mechanism in stressful situations, especially in health-related problems. It controls the mind and gives meaning and hope. It helps people to find coping strategies and have a positive outlook on life after death.(14) The association between spirituality and religion cannot be neglected.(15) Religion can affect the burden and the level of stress and anxiety in caregivers of patients.(16) Researchers have acknowledged that religious coping is effective for enhancing illness adjustment. Religious coping refers to involvement of religious beliefs and attitudes in problem-solving and control of difficult situations by preventing or reducing negative psychological consequences.(6) It is shown that religious and spiritual beliefs play an important role in caregiver adaptation with difficult situation of caring for stroke patients.(17)

Spiritual-religious coping reduced the level of depression, anxiety, and stress in caregivers of children with leukemia.(18) Given the anxiety of relatives of CCU patients, focus of the treatment team on the patient, and negligence of mental state of caregivers, the present study aimed to determine the level of anxiety and of relatives of CCU patients its association with spiritual health and religious coping.

## Methods

This was a descriptive, correlational study. The required data was collected from August 2018 to October 2018. All relatives of patients admitted to CCUs of the hospitals affiliated with the Jahrom University of Medical Sciences (in Iran) were studied. The sample size was calculated as 130 given type I error of 0.05, type II error of 0.2, and correlation of spiritual health with anxiety scores in older patients admitted to CCU (r = 0.243) as found in a previous study (12). The sample size was increased to 300 to recover the possible loss and enhance accuracy of the study. All relatives of CCU patients meeting the inclusion criteria were selected using convenience sampling. Since the number of CCU beds was identical in the hospitals, equal number of samples were selected from two hospitals in Jahrom. Inclusion criteria were willingness to participate in the study, minimum standard of literacy, 24 to 48 hours after admission to CCU, a close family member (spouse, child, father, mother, sister, and brother), and 18-65 years of age. Exclusion criteria were intake of anxiolytic and sedative drugs, history of diagnosis with a cognitive and psychological disorder, and uncompleted questionnaires.

A demographic questionnaire, the Paloutzian-Ellison Spiritual Well-Being Scale (SWBS), the Spielberger State-Trait Anxiety Inventory (STAI), and the Pargament Brief RCOPE questionnaire were used for data collection. It was validated for the local setting. The demographic questionnaire collected data on patients’ relatives (age, gender, occupation, education, relationship with the patient, marital status, number of children) and the patient (marital status, number of children, age, gender, occupation, medical diagnosis, date of admission to CCU, date of hospitalization, and medical history. The SWBS contains 20 items and measures two subscales. The odd items measure the religious well-being subscale (experience of a satisfying relationship with God, and the even items measure the existential well-being subscale (a purposeful life and life satisfaction). A six-point Likert scale is used to rate the items ranging from “1 = strongly agree” to “6 = strongly disagree.” The negative items are reverse-scored. The scores of religious and existential well-being range from 10 to 60. The higher the score, the greater the religious and existential health. The scores of spiritual well-being range from 20 to 120. The spiritual health is categorized into categories of low level (20-40), moderate level (41-99), and high level (100-120). Balanjani *et al.* calculated Cronbach’s alphas of the spiritual well-being scale, religious well-being subscale, and existential well-being subscale as 0.92, 0.86, and 0.87, respectively.(19)

STAI contains two subscales of state anxiety and trait anxiety scoring from 1 to 4 (from very low to very high). The anxiety scores range from 20 to 80; state-trait anxiety scores of 20-31, 32-42, 43-53, 54-64, 65-75, and >75 indicate mild anxiety, moderate to low anxiety, moderate to high anxiety, relatively severe anxiety, severe anxiety, and strongly severe anxiety, respectively. Trait anxiety scale is scored from 1 to 4 (almost never, sometimes, often, and almost always). The Persian version of STAI was used in various studies in Iran. Its content validity and reliability were confirmed through Cronbach’s alpha calculation (0.85 to 0.93).(20) The Brief RCOPE questionnaire contains 14 items scoring based on a four-point Likert scale ranging from 1 to 4. Items 1-7 measure positive behaviors and items 8-14 measure negative behaviors. Its subscales are benevolent religious reappraisals, collaborative religious coping, seeking spiritual support, religious purification, and religious forgiveness. Aflakseir and Colman confirmed its validity and assessed its reliability through Cronbach’s alpha of 0.86.(21) Data were analyzed in SPSS 22 through descriptive statistics to describe the studied variables as well as Spearman correlation coefficient and linear regression at the significance level of 0.05.

The project was approved by the Ethics Committee of the Shiraz University of Medical Sciences (Code of Ethics = IR.SUMS.REC.1397.608). The participants were ensured of data confidentiality and the right to leave the project whenever they desired at any phase of the study.

## Results 

The findings showed that the majority of the patients’ relatives were female (63.3%), married (83.3%), housewives (48.7%), in the age range of 31-45 (45%), with a high school diploma (56%), and 1-3 children (52%). The majority of patients were 61-91 years old (50.6%), female (53.3%), married (97.8%), with 4-6 children (37.8%). In addition, 56.1%, 67.8%, and 78.3% of patients had unstable angina, 1-3 hospitalization episodes, and a history of disease, respectively.

The mean scores of total anxiety, state anxiety, and trait anxiety in the relatives were 88.75±16.67, 45.02±10.26, and 43.72±8.35, respectively. Anxiety of relatives was high level. State anxiety was moderate in 71.8% of patients and Trait anxiety was moderate in 77.6% (Table 1). The mean score of total spiritual health was 106.23±13.09. Spiritual health level was high in 76.7% of relatives and moderate in 23.3% of them.

**Table 1:** Anxiety score of relatives of CCU patients

Anxiety level	% State anxiety	% Trait anxiety
Mild	7	6
Moderate to low	36.3	40.3
Moderate to high	35.3	37.3
Relatively severe	18	15
Severe	3	1.3
Strongly severe	0.3	0

Anxiety score had an inverse relationship with spiritual health and a direct relationship with negative religious coping score (r=0.25). However, no significant relationship was found between total anxiety score and positive religious coping (Table 2).

**Table 2:** Correlation of Anxiety and its subscales with Spiritual Health and its subscales in relatives of CCU patients

Spiritual health Anxiety	Total	Religious Health	Existential Health	Positive Coping	Negative Coping
Total	r = -0.52*	r= -0.33*	r = -0.55*	r = 0.034**	r = 0.25*
State Anxiety	r = -0.49*	r = -0.3*	r = -0.52*	r = 0.04**	r = 0.2*
Trait Anxiety	r = -0.45*	r = -0.29*	r = -0.47*	r = 0.04**	r = 0.25*

*: *p*<0.001; **p>0.05

Spearman correlation test results showed a positive significant relationship between religious coping score and spiritual health in the relatives. The relationship of spiritual health and its subscales (religious and existential well-being) with positive religious coping was also significant and positive. There was also an inverse significant correlation between negative coping score and overall spiritual health score (and its religious and existential subscales). (Table 3)

**Table 3:** Correlation of religious coping with spiritual health and its subscales in relatives of CCU patients

Score	Positive Coping	Negative Coping
Total Spiritual Health	r = 0.19*	r = -0.36*
Religious well-being	r = 0.24*	r = -0.42*
Existential well-being	r = 0.14*	r = 0.31*

*: *p*<0.001

The results of regression analysis showed that one-unit increase in spiritual well-being score decreased anxiety score by 0.69 unit. Since coefficient of determination (R^2^) was 0.29, the spiritual health score can explain 29% of changes in relatives’ anxiety score. The remaining changes are caused by unknown factors not included in the model. One-unit increase in negative coping score increased anxiety score by 1.26 unit. Since the coefficient of determination (R^2^) was 0.1, the negative coping score can explain 10% of changes in anxiety scores of relatives (Table 4).

**Table 4:** Linear regression analysis to assess the predictor role of spiritual health and religious coping on anxiety of relatives

	Model	Unstandardized coefficient	Unstandardized coefficient	Beta	t	***p-*value**
		B	SE			
Spiritual health as predictor of anxiety in relatives	Constant	162.11	6.64	-	24.4	0.001
Spiritual health as predictor of anxiety in relatives	Spiritual Health	-0.69	0.06	-0.54	11.13	0.001
Religious coping as predictor of anxiety in relatives	Constant	64	23.38	-	2.74	0.007
Religious coping as predictor of anxiety in relatives	Positive Coping	0.41	0.84	0.03	0.49	0.62
Religious coping as predictor of anxiety in relatives	Negative Coping	1.26	0.22	0.32	5.75	0.001

## Discussion

A family member usually accompanies the patient as he/she is admitted to the CCU. The accompanier acts as a link between the family and the treatment team and they are as important as the patient in terms of paying attention. The results of the present study showed that anxiety score of relatives of patients admitted to CCU was very high. The level of anxiety was moderate to high in more than one-third of the patients’ relatives. This result was consistent with the results of the study by Famis *et al.*(22) who showed that anxiety of the relatives varied from 35% to 45%. Lacerda *et al.*(23) found anxiety in 77% of patients’ relatives but Bolosi *et al.*(4) found anxiety in 13.9% of patients, which was lower than the rate of anxiety in the present study. 

Relatives of patients admitted to critical care units experience a significant level of anxiety due to various causes such as the stressful environment, risk of death for patients, and negligence of relatives’ needs (6). Most ICU admissions are unplanned and urgent, which contribute to severe anxiety of the family members.(24) Spiritual health of relatives of CCU patients was high in the present study. This result was consistent with the results of the study by Heidari *et al.* on relatives of acute patients.(25) The sense of spirituality controls minds, gives meaning and hope, helps to find efficient strategies and have positive outlook.(19) The results of the present study showed that the mean score of positive religious coping was high and the mean score of negative religious coping was low in relatives of patients. Iran is a religious country that fosters spiritual beliefs, Therefore, patients’ relatives rely on their religious beliefs to cope with stressful situations and reinforce religious coping to care for their beloved ones.(10) Similar to the present study, Pearce *et al.* assessed religious coping in caregivers of mentally ill patients and reported high level of religious coping.(26) 

However, Tambri *et al.* reported low level of religious coping in caregivers of cancer patients and religious coping was neglected in caring for these patients.(27) Religion helps them to be calm and overcome their problems.(19) Similar to the present study, Hedayati *et al.,*(13) showed that as the scores of spiritual health and its subscales increased, scores of total anxiety, state anxiety, and trait anxiety significantly decreased. Religious beliefs and spirituality help people to cope with stress, give them hope, foster a positive outlook, enhance the inner peace and help them to adopt efficient strategies to cope with stress.(26) Spiritual health and religious practices are viewed as a useful coping approach in the psychology and play an effective role in improving the quality of patient care received from the relatives.(16)The results of the present study found no relationship between positive religious coping and total anxiety score and its subscales (state and trait anxiety) in relatives of patients. However, anxiety of patients’ relatives significantly increased as the score of negative coping increased. Negative coping explained 10% of changes in relatives’ anxiety scores. Francis *et al.* showed that negative religious coping was associated with symptoms of depression and anxiety in medical students.(28) Chong Guan *et al.* assessed the relationship of religion and religious coping with anxiety and depression. They showed that patients with anxiety and depression were more likely to use negative adjustment and had unorganized religion.(29) Strong relationship between family members in the Iranian society help them to care for their patients in case that one of their family members became ill.(30) 

Religious coping help people to endure difficulties and prevent anxiety. Prayers, reciting Quranic verses, or attending religious ceremonies help religious people to be less vulnerable to stressful life events. Health care providers can reinforce spiritual health and religious coping of relatives to improve care services and relieve the stress of disease.(16) Limitations of the study included lack of cooperation of some relatives due to their mental state and physical exhaustion that might affect accuracy of the study.

## Conclusion

The findings of the present study indicate a very high level of anxiety and a high level of spiritual well-being and religious coping in relatives of CCU patients. Given the inverse relationship of spiritual well-being score and its subscales with total anxiety score, state anxiety, and trait anxiety, reinforcing religious approaches that rely on religious beliefs and coping and spiritual well-being, as well as using religious and spiritual interventions can be beneficial in reducing the anxiety of relatives of CCU patients.

## References

[1] Pfeffer MA (2017). Heart Failure and Hypertension: Importance of Prevention. Med. Clin. North Am..

[2] Morton PG, Fontaine DK (2013). Essentials of Critical Care Nursing A Holistic Approach: Wolters Kluwer Health| Lippincott Williams & Wilkins.

[3] Sadeghi M, Haghdoost AA, Bahrampour A, Dehghani M (2017). Modeling the Burden of Cardiovascular Diseases in Iran from 2005 to 2025: The Impact of Demographic Changes. Iran. J. Public Health..

[4] Bolosi M, Peritogiannis V, Tzimas P, Margaritis A, Milios K, Rizos DV (2018). Depressive and anxiety symptoms in relatives of intensive care unit patients and the perceived need for support. J. Neurosci. Rural Pract..

[5] Wartella JE, Auerbach SM, Ward KR (2009). Emotional distress, coping and adjustment in family members of neuroscience intensive care unit patients. J. Psychosom. Res..

[6] Alexandri A, Georgiadi E, Mattheou P, Polikandrioti M (2017). Factors associated with anxiety and depression in hospitalized patients with first episode of acute myocardial infarction. Arch. Med. Sci. Atheroscler. Dis..

[7] Pashaei F, Taleghani F, Tavakol K, Rezaei AE (2010). Family experiences from caregivering of patient with coronary artery bypass graft surgery: a qualitative study. Ira. J. Nurs. Res..

[8] van Beusekom I, Bakhshi-Raiez F, de Keizer NF, Dongelmans DA, van der Schaaf M (2016). Reported burden on informal caregivers of ICU survivors: a literature review. Crit. Care (London, England).

[9] Konstanti Z, Gouva M, Dragioti E, Nakos G, Koulouras V (2016). Symptoms of Cardiac Anxiety in Family Members of Intensive Care Unit Patients. Am. J. Crit. Care..

[10] Momennasab M, Moattari M, Abbaszadeh A, Shamshiri B (2013). Spiritual experience of heart attack patients: A qualitative study. J. Qual. Res. Health Sci..

[11] Gaur KL, Sharma M (2014). Measuring Spiritual Health: Spiritual Health Assessment Scale (SHAS). Iran. J. Innov. Res. Dev..

[12] Craven R, Hirnle C, Jensen S (2003). Fundamental of nursing: Human health and function.

[13] Hedayati E, Hazrati M, Momen Nasab M, Shokoohi H, Afkari F (2016). The Relationship Between Spiritual Well-being and Anxiety of Aged People Admitted in Coronary Care Units. Iran. J. Ageing..

[14] Baldacchino D (2015). Spiritual care education of health care professionals. Religions.

[15] Platovnjak I (2017). The relationship between spirituality, religion, and culture. Studia Gdańskie.

[16] Hatefi M, Vaisi-Raygani A, Borji M, Tarjoman A (2020). Investigating the Relationship between Religious Beliefs with Care Burden, Stress, Anxiety, and Depression in Caregivers of Patients with Spinal Cord Injuries. J. Relig. Health..

[17] Gholamzadeh S, Hamid TA, Basri H, Sharif F, Ibrahim R (2014). Religious coping and psychological well-being among Iranian stroke caregivers. Iran J. Nurs. Midwifery Res..

[18] Zafarian Moghaddam E, Behnam Vashani HR, Reihani T, Namazi Zadegan S (2016). The Effect of Spiritual education on depression, anxiety and stress of caregivers of children with leukemia. J. Torbat Heydariyeh Univ. Med. Sci..

[19] Baljani E, Khashabi J, Amanpour E, Azimi N (2011). Relationship between Spiritual Well-being, Religion, and Hope among Patients with Cancer. Hayat.

[20] Bastani F, Ali Abadi T, Haghani H (2012). The Effectiveness of Participatory Care Program in Neonatal Intensive Care Unit on State Anxiety of Mothers of Preterm Newborns. JBUMS.

[21] Aflakseir A, Coleman PG (2011). Initial development of the Iranian religious coping scale. J. Muslim Ment. Health..

[22] Fumis RRL, Ranzani OT, Faria PP, Schettino G (2015). Anxiety, depression, and satisfaction in close relatives of patients in an open visiting policy intensive care unit in Brazil. J. Crit. Care..

[23] Lacerda MS, Cirelli MA, Barros ALBLd, JdL Lopes (2017). Anxiety, stress and depression in family members of patients with heart failure. Rev. Escol. Enferm.USP.

[24] (2012). Loiselle CG, Gélinas C, Cassoff J, Boileau J, McVey L. Intensive and Crit. Care Nurs.

[25] Heidari J, Jafari H, Janbabaei G (2015). Life Quality Related To Spiritual Health And Factors Affecting It In Patients Afflicted By Digestive System Metastatic Cancer. Mater Sociomed..

[26] Pearce MJ, Medoff D, Lawrence RE, Dixon L (2016). Religious Coping Among Adults Caring for Family Members with Serious Mental Illness. Community Ment. Health J..

[27] Thombre A, Sherman AC, Simonton S (2010). Religious coping and posttraumatic growth among family caregivers of cancer patients in India. J. Psychosoc. Oncol..

[28] Francis B, Gill JS, Yit Han N, Petrus CF, Azhar FL, Ahmad Sabki Z (2019). Religious Coping, Religiosity, Depression and Anxiety among Medical Students in a Multi-Religious Setting. Int. J. Environ. Res. Public Health..

[29] Ng Chong Guan, Mohamed S, Sulaiman AH, Zainal NZ (2017). Anxiety and Depression in Cancer Patients: The Association with Religiosity and Religious Coping. J. Relig. Health..

[30] Ebrahimi H, Hasankhani H, Namdar H, Khodadadi E, Fooladi M (2017). Dealing with Chronic Illness: Experiences of Iranian Families of Persons with Multiple Sclerosis-A Qualitative Study. Mult. Scler. Int..

